# Prognosis of acute low back pain: design of a prospective inception cohort study

**DOI:** 10.1186/1471-2474-7-54

**Published:** 2006-06-22

**Authors:** Nicholas Henschke, Christopher G Maher, Kathryn M Refshauge, Robert D Herbert, Robert G Cumming, Jane Bleasel, John York, Anurina Das, James H McAuley

**Affiliations:** 1 Back Pain Research Group, School of Physiotherapy, University of Sydney, PO Box 170 Lidcombe, NSW 1825, Australia; 2 School of Public Health, University of Sydney, Australia; 3 School of Physiotherapy, University of Sydney, Australia; 4 Department of Rheumatology, Royal Prince Alfred Hospital, Sydney, Australia

## Abstract

**Background:**

Clinical guidelines generally portray acute low back pain as a benign and self-limiting condition. However, evidence about the clinical course of acute low back pain is contradictory and the risk of subsequently developing chronic low back pain remains uncertain. There are few high quality prognosis studies and none that have measured pain, disability and return to work over a 12 month period. This study aims to provide the first estimates of the one year prognosis of acute low back pain (pain of less than 2 weeks duration) in patients consulting primary care practitioners. A secondary aim is to identify factors that are associated with the prognosis of low back pain.

**Methods/Design:**

The study is a prospective inception cohort study. Consecutive patients consulting general medical practitioners, physiotherapists and chiropractors in the Sydney metropolitan region will complete a baseline questionnaire regarding their back pain. Subsequently these patients will be followed up by telephone 6 weeks, 3 months and 12 months after the initial consultation. Patients will be considered to have recovered from the episode of back pain if they have no pain and no limitation of activity, and have returned to pre-injury work status. Life tables will be generated to determine the one year prognosis of acute low back pain. Prognostic factors will be assessed using Cox regression.

**Discussion:**

This study will provide the first estimates of the one year prognosis of acute low back pain in a representative sample of primary care patients.

## Background

It is widely agreed that acute low back pain is common, can be seriously disabling, and imposes an enormous social and economic burden on the community [1]. To improve the management of this condition clinical practice guidelines have been developed in at least 12 countries. In general the guidelines provide similar information [2]. A common theme is that acute low back pain should be managed in primary care because it is generally benign: recovery tends to be both rapid and complete, and the few cases of serious disease can be readily detected with a clinical assessment [2].

While most of the low back pain guidelines review the evidence prior to making recommendations for therapy, few have adopted an evidence-based approach to making recommendations on prognosis. The guidelines often do not refer to original research but instead have relied upon narrative review papers or previous guidelines. For example the commonly cited statistic on prognosis in the 2000 UK Guideline [3] "90% recover within 6 weeks" is attributed to page 26 of the 1996 UK guideline [4], even though the 1996 guideline provides no source for this information. Despite the lack of supporting evidence [5], this statistic is still widely reported. Without a comprehensive understanding of the clinical course of low back pain, clinicians will be unable to provide accurate information to patients regarding their prognosis [5].

Although most current guidelines suggest a favourable prognosis, recent systematic reviews suggest that while patients with an acute episode of low back pain may improve rapidly, the risk of developing chronic low back pain (i.e. pain persisting longer than 3 months) is uncertain [5,6]. Studies have reported estimates of the risk of developing chronic low back pain that range from 2% [7] to 56% [8]. This inconsistency has been attributed to methodological shortcomings of the prognostic studies or to the recruitment of an unrepresentative cohort of low back pain patients [6,9]. It may also be due to heterogeneity in outcome measurements used in these studies [10]. One systematic review on the prognosis of acute low back pain [6] found 15 studies which met the inclusion criteria, but only two of the studies reported data beyond the 3 month follow-up. The same review found only three studies which reported on prognostic factors for at least 80% of the sample. No study measured pain, disability, or return to work in a primary care setting and followed patients for one year.

The notion that acute low back pain has a favourable prognosis, a view common to all guidelines, should be reconsidered because of the inconsistency in the outcomes reported and the lack of long-term follow-up data. Acute low back pain may not be a benign, self-limiting condition. Our paper reports the design of a study which will determine the medium-term (1 year) prognosis for people with acute low back pain presenting to community primary care providers (general medical practitioners, physiotherapists and chiropractors). The outcomes of interest will be time to recovery, which is defined by measures of pain, disability and work status. A secondary aim is to develop a prognostic model of acute low back pain.

## Methods/Design

The study will be an inception cohort study. It is part of a larger cohort study which will also assess the accuracy of the diagnostic triage for detecting serious spinal pathology in patients presenting with acute low back pain. Only the prognosis component of the study is described here.

The target population is patients with acute low back pain who consult practitioners from three major primary care professions, namely chiropractors, physiotherapists and general medical practitioners. Consecutive patients will be invited to complete a baseline assessment and have a series of three follow-up assessments performed via telephone calls over a 12-month period.

### Study population

A cohort of 1,000 subjects with acute low back pain will be recruited from the Sydney metropolitan region. Data from the 2001 Australian Census will be used to characterize the socioeconomic levels of postcode (zipcode) areas within the Sydney Metropolitan region. A number of postcode areas will be recruited to the study in order to achieve a range of socio-economic levels.

All practitioners within the study area will be invited to participate in the study. Practitioner names and clinic addresses will be extracted from telephone directories, professional registry listings and through professional associations of the three practitioner groups. Every practitioner identified within the study area will be sent a letter of invitation and a reply paid postcard, enabling them to indicate whether they intend to participate in the study, or whether they require further information. Three weeks after the letters are mailed out, all practitioners who have not replied via postcard will be contacted by telephone and invited to participate in the study. Practitioners will be excluded if they are not current primary care providers, e.g. specialists or retired practitioners, are not practising within the study area, or the practitioner details are not sufficient to contact them regarding the study. A record will be kept of practitioners who choose not to participate and where given, the reasons for their decisions.

Participating practitioners will be trained in either small groups or individual sessions. Training involves an explanation of the purpose and methods of the study and instruction on how to perform a standardized diagnostic triage. The practitioners will be asked to identify all eligible patients presenting at their clinics, assist patients to complete the baseline questionnaire, record the results of 25 clinical assessment "red flags", and record their diagnoses based on the AHCPR clinical practice guidelines [11]. Practitioners will also be given a copy of current guidelines for the management of acute low back pain and asked to follow them when appropriate.

### Inclusion and exclusion criteria

**Figure 1:**
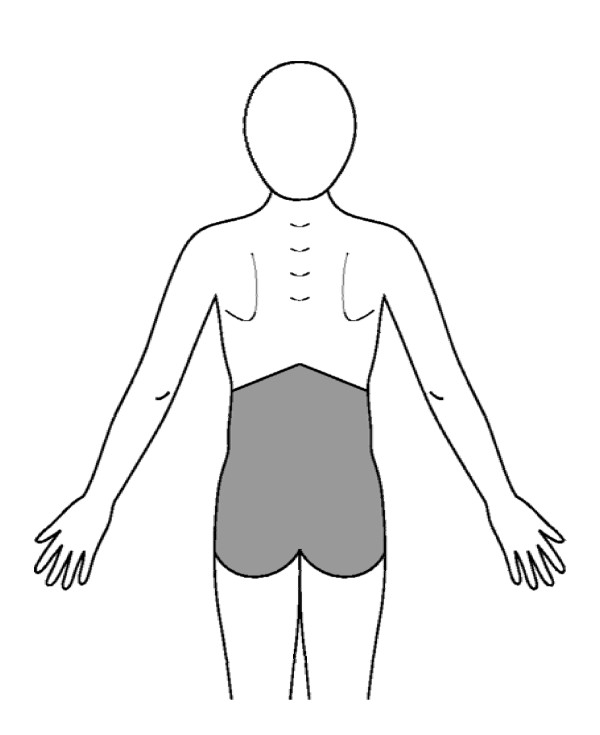
Spatial location of low back pain [13].

Participating practitioners will be asked to screen all patients with the primary complaint of low back pain who present to their clinics. To be eligible for inclusion, patients must consult one of the practitioners for management of an episode of acute low back pain. We have used the definition for an episode of acute low back pain proposed by de Vet and colleagues [12]. This is defined by pain in the area bounded superiorly by T12 and inferiorly by the buttock crease [13] (Figure 1), lasting for more than 24 hours but less than two weeks, and preceded by a period of at least one month without back pain [12]. Participants remain eligible if they have pain referred beyond this region. To be included, participants must be at least 14 years old, provide written consent to participate in the study, and be able to speak and read English. Potential subjects will be excluded if a serious pathology (e.g. cancer, spinal infection, spinal fracture, inflammatory disorder) has already been diagnosed as the cause of this episode of low back pain prior to the presentation of the patient in primary care.

A record will be kept of patients who choose not to participate and their reasons for doing so, as well as patients with back pain who are ineligible to participate, and the reasons for exclusion. Recruitment will continue at all sites from the date of training until the target sample of 1,000 participants is achieved. With this sample size, the 95% confidence interval for an observed proportion of 5% extends from 3.8% to 6.5%, and the 95% confidence interval for an observed proportion of 50% extends from 46.9% to 53.1%. Practitioners will be contacted every two weeks to ensure that they are adhering to the study protocol. Also, practitioners will be reminded to recruit all eligible patients so that the cohort consists as far as possible of a consecutive sample.

### Baseline measures

Baseline data will be used to comprehensively describe the inception cohort and test putative predictors of prognosis. The specific classes of predictors being measured are socio-demographic characteristics, general health, previous history, and psychological characteristics (see additional file 1).

As there is no universally accepted single measure of recovery from low back pain, we will sample three dimensions of recovery: pain intensity, disability due to pain, and work status. The first two questions are adaptations of items 7 and 8 of the SF-36 [14]: "How much low back pain have you had in the past week?", and "During the past week, how much did low back pain interfere with your normal work (including both work outside the home and housework?)". The original wording was changed from 'bodily pain' to 'low back pain' to reflect our specific interest in low back pain. The subset of participants employed at the time of onset of symptoms will be asked to rate their work status on a 9 point scale adapted from the scale developed by Kenny [15].

### Follow-up procedure

Completed assessment booklets will be collected every two weeks from study practitioners by a research assistant. When the booklets have been retrieved, the data concerning the participant contact details, assessment date and baseline data will be entered into a database. Any missing or conflicting data from the assessment booklet will be checked immediately, with every attempt made to contact the participant as soon after collection of the booklet as possible. Follow-up assessments will be conducted 6 weeks, 3 months and 12 months after the initial assessment.

At each follow-up time point, participants will be asked whether they have become pain-free, have no disability due to back pain, and returned to their pre-injury work status. Only when participants report achieving one of these points and remained there for a whole month, are they considered "recovered" in this dimension at the beginning of that month [12]. Participants will also be asked about their current status of pain, function, and work status and whether a serious spinal pathology has been diagnosed as the cause of their low back pain episode.

For participants, there will be a A$10 (incl. GST) compensation for time and inconvenience for each of the telephone follow-ups with an additional A$10 (incl. GST) payment if all telephone follow-ups are completed, to be paid following the 12 month follow-up. Practitioners will also be compensated A$50 (incl. GST) for each eligible patient enrolled, and A$10 (incl. GST) for each patient who is screened but ineligible.

Several mechanisms will be used to ensure study data are of high quality. First, participating practitioners will be trained in the trial protocol and the Agency for Health Care Policy and Research (AHCPR) clinical assessment [11] in a standardised format. To maximise compliance with the clinical assessment, summaries of the AHCPR examination will be provided for display in each clinic. The research assistant who collects the data sheets will provide feedback to practitioners if there is evidence that the protocol is not being followed. Data will be entered and double checked by two people, and inconsistencies resolved by contacting the participant where appropriate, or via consensus.

### Data analysis

Data will be collected on when people return to pre-injury work status and/or had no disability and/or had no pain, to enable construction of life tables. The life tables will be used to describe the prognosis of patients with non-specific low back pain presenting to primary care practitioners. Median survival times (days to recovery) will be determined for each of the three recovery measures.

Cox regression will be used to evaluate putative prognostic factors. The independent variables for the regression will be chosen from among those collected at baseline (see additional file 1). A correlation matrix will be inspected to determine relationships between candidate variables and outcome. Variables with strong correlations (p < 0.10) will be identified and entered into the regression model.

Linear regression will be used to predict continuous outcomes such as days off work. If necessary, dependent variables will be transformed so that they satisfy assumptions of normality of residuals and heteroscedasticity. If there is evidence of non-linear effects, quadratic or higher order terms will be added to the regression model.

## Discussion

Patients with acute low back pain of less than 2 weeks duration will be recruited from the three primary care professions who most frequently manage low back pain in Australia. This will provide a representative cohort of patients to allow better provision of prognostic information to primary care providers. The choice of outcome measures reflects a standardised definition for an episode of low back pain [12] proposed to lead to more uniform reporting of the course of low back pain. By measuring three dimensions of recovery (pain, disability, and work status), a complete description of the impact of low back pain can be determined.

This study has been designed to include key methodological features that have been recognised as minimising bias in prognostic studies. These features include sampling of a representative inception cohort with a high rate of follow-up [16]. The proposed study will provide the first estimate of the one year prognosis of acute low back pain, measured in terms of pain, disability, and return to work, for patients presenting to primary care.

## Competing interests

The author(s) declare that they have no competing interests.

## Authors' contributions

All authors participated in the design of the study. NH drafted the manuscript with input from the other authors. All authors read, revised and approved the final manuscript.

## Pre-publication history

The pre-publication history for this paper can be accessed here:
